# An Upstream Open Reading Frame Controls Translation of *var2csa*, a Gene Implicated in Placental Malaria

**DOI:** 10.1371/journal.ppat.1000256

**Published:** 2009-01-02

**Authors:** Borko Amulic, Ali Salanti, Thomas Lavstsen, Morten A. Nielsen, Kirk W. Deitsch

**Affiliations:** 1 Department of Microbiology and Immunology, Weill Medical College of Cornell University, New York, New York, United States of America; 2 Centre for Medical Parasitology at Department of International Health, Immunology, and Microbiology, University of Copenhagen, Department of Infectious Diseases, Copenhagen University Hospital (Rigshospitalet), Copenhagen, Denmark; Swiss Tropical Institute, Switzerland

## Abstract

Malaria, caused by the parasite *Plasmodium falciparum*, is responsible for substantial morbidity, mortality and economic losses in tropical regions of the world. Pregnant women are exceptionally vulnerable to severe consequences of the infection, due to the specific adhesion of parasite-infected erythrocytes in the placenta. This adhesion is mediated by a unique variant of PfEMP1, a parasite encoded, hyper-variable antigen placed on the surface of infected cells. This variant, called VAR2CSA, binds to chondroitin sulfate A on syncytiotrophoblasts in the intervillous space of placentas. VAR2CSA appears to only be expressed in the presence of a placenta, suggesting that its expression is actively repressed in men, children or non-pregnant women; however, the mechanism of repression is not understood. Using cultured parasite lines and reporter gene constructs, we show that the gene encoding VAR2CSA contains a small upstream open reading frame that acts to repress translation of the resulting mRNA, revealing a novel form of gene regulation in malaria parasites. The mechanism underlying this translational repression is reversible, allowing high levels of protein translation upon selection, thus potentially enabling parasites to upregulate expression of this variant antigen in the presence of the appropriate host tissue.

## Author Summary

Infection by the protozoan parasite *Plasmodium falciparum* results in the most severe form of human malaria and is responsible for significant morbidity and mortality in the developing world. This disease can be particularly severe in pregnant women due to the specific adhesion of parasite-infected red blood cells within the placenta. Expression of a single gene called *var2csa* has been linked to targeting of the placenta, and thus this gene represents a key element in the virulence of *P. falciparum* infections. It was previously shown that *var2csa* is predominantly expressed by parasites in pregnant women, suggesting that parasites might have the ability to down regulate this gene when no placenta is available. Here we describe an upstream open reading frame (uORF)–mediated mechanism used by parasites to repress translation of *var2csa* mRNA, thus providing a mechanism for controlling gene expression at the level of protein translation. This mechanism has not previously been observed in malaria parasites, and may represent a form of regulation used to control expression of other genes within the genome.

## Introduction

Adults living in areas endemic for *Plasmodium falciparum* malaria acquire partial immunity through repeated infections. This immunity is suddenly lost with the onset of a first pregnancy, resulting in frequent occurrence of pregnancy associated malaria (PAM), a form of the disease which endangers both the mother and the fetus [1]. Susceptibility to PAM is parity dependent; primigravid women are most severely affected and the likelihood of severe complications lessens with subsequent pregnancies [2]. Red blood cells infected with *P. falciparum* adhere to various host receptors in the vasculature in order to avoid clearance by the spleen [3], and this adherence causes several distinct disease syndromes, most notably cerebral malaria resulting from adherence of parasites in the brain. Parasites causing PAM are functionally and immunologically unique, primarily because they bind to the proteoglycan chondroitin sulphate A (CSA) in the placenta rather than the typical receptors associated with adherence in other tissues such as CD36 and ICAM [4]. Binding to the syncytiotrophoblasts of the placenta results in massive sequestration of infected erythrocytes (IEs) in this organ [5].

Cytoadhesion of infected erythrocytes is mediated by parasite-encoded variant surface antigens collectively called PfEMP-1. Accumulation of antibodies that recognize a large proportion of the polymorphic PfEMP-1 family is considered an important aspect of acquisition of natural immunity against malaria [6],[7]. These proteins are encoded by the *var* multigene family [8] and undergo mutually exclusive transcription [9], ensuring that each parasite only produces one antigen at a time. Continuous switching of transcription to different *var* genes allows avoidance of a successful immune response thus enabling the establishment of a chronic infection [10]. A unique *var* gene, called *var2csa*, encodes the PfEMP-1 molecule that binds chondroitin sulfate A (CSA) [11]–[13]. The genome of a typical parasite contains a repertoire of approximately 60 *var* genes which vary between field isolates; *var2csa* is one of the few genes that appear to be conserved and found in most, if not all, parasite isolates. The selective pressure acting on preservation of this gene is further exemplified by the fact that a homologue is even found in the chimpanzee parasite *Plasmodium reichenowi* [14].

While antibodies against most forms of PfEMP1 are acquired by early adulthood in individuals growing up in malaria endemic regions, antibodies specific to the CSA-binding variant seem to be acquired in a sex-specific and parity-dependent manner. With the exception of one study [15], highly reactive antibodies were only detected in women who have been pregnant [16]–[19], with levels of reactivity increasing with increasing numbers of pregnancies [12],[20]. This led to hypothesis that *var2csa* might be subject to a unique regulatory mechanism that does not apply to the rest of the *var* gene family. It has also been suggested that it might even be specifically upregulated during pregnancy [21]. *var2csa* transcripts can, however, be detected in non-pregnant individuals, including children and men [22]–[24], suggesting post-transcriptional repression mechanisms might be operating in these individuals, resulting in lower exposure to the immune system. Contrary evidence was provided by a proteomic study which detected VAR2CSA in parasites of non-placental origin [25] as well as an earlier study that demonstrated CSA-binding activity of parasites from non-pregnant hosts [26]. Nevertheless, a recent report by Mok et al. demonstrated post-transcriptional repression of *var2csa* in clones of cultured parasites that were actively transcribing this variant [27].

*var2csa* has a unique 5′ regulatory region (UpsE) that cannot be categorized into groups established for other *var* genes. The 5′ untranslated region of the mRNA contains a 360 bp upstream open reading frame (uORF) that ends 269 bp 5′ of the translational start site [28]. This uORF is conserved across *P. falciparum* isolates and in *P. reichenowi*, indicating that it is under similar selection pressure as the rest of the gene. uORFs have recently emerged as novel regulators of eukaryotic translation [29] with some estimates predicting that they are found in up to half of human and rodent 5′ leader sequences [30]. They act as repressors of protein synthesis via several different mechanisms, including stalling of the ribosome [31], prevention of re-initiation [32] and induction of nonsense mediated decay [33], thus acting as repressors of protein expression. The presence of a uORF in the 5′ untranslated region of *var2csa* raises the possibility that this element might function to prevent translation of *var2csa* mRNA in situations where the ligand for the encoded PfEMP1 is not available, for instance in the absence of a placenta.

Here we test the hypothesis that the *var2csa* uORF functions as a repressor of mRNA translation. Using reporter gene constructs in both transiently transfected and stably transformed parasites, we show that the uORF does indeed repress mRNA translation, but that it is possible to select parasites that have reversed this repression and actively translate the mRNA. Further, cultured parasites were obtained that similarly displayed translational repression of *var2csa* expression, indicating that this regulatory mechanism also occurs during expression of the endogenous gene. These data suggest that an additional level of regulation controls expression of the PfEMP1 variant associated with pregnancy associated malaria, perhaps to prevent its expression at times when no placenta is available for binding.

## Results

### The *var2csa* uORF acts as a repressor of reporter gene expression

**Figure 1:**
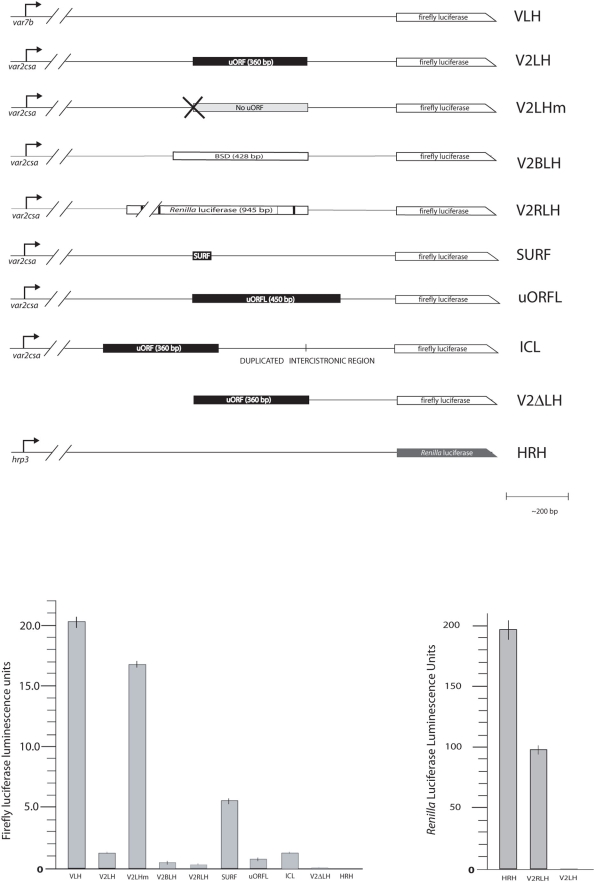
The *var2csa* uORF is a repressive element. A. Constructs used in transient transfections. Constructs drive expression of either the firefly *luciferase* reporter gene, *Renilla luciferase* (in the case of control plasmid HRH) or both (in the case of V2RLH). VLH contains the un-modified promoter and upstream regulatory region from *var7b*. V2LH contains the un-modified promoter and upstream regulatory region from *var2csa*. V2LHm is identical to V2LH, except a single base pair mutation has altered the start codon of the upstream open reading (uORF) from ATG to ACG. In V2BLH, the uORF has been replaced with the *bsd* coding region while in V2RLH the uORF has been replaced by the coding region for *Renilla luciferase*. In V2ΔLH the entire region upstream of the uORF, including the transcription start site, has been deleted. In SURF the uORF has been shortened to 48 bp by the introduction of a premature stop codon, while in uORFL it has been lengthened to 450 bp by eliminating the endogenous stop codon. ICL contains the intact upstream regulatory region, including the uORF, however the intercistronic region has been duplicated. B. Levels of firefly luciferase expression from each construct shown in A. C. Levels of *Renilla* luciferase expression. V2RLH supports translation of robust levels of *Renilla* luciferase indicating that the uORF can be translated. The plasmid HRH, containing the strong *hrp3* promoter, was employed as a positive control for *Renilla* luciferase expression, while V2LH, which does not contain the *Renilla* luciferase gene, was a negative control. All assays were done simultaneously in triplicate.

Analysis of the 5′ leader sequence of *var2csa* transcripts from parasites selected for CSA binding, as well as parasites isolated from pregnant women, previously revealed that the uORF found upstream of the PfEMP1 coding region is not spliced out and is in fact present in the mRNA [28], indicating that VAR2CSA is encoded by a bicistronic transcript. In order to determine the effect of this uORF on gene expression, the promoter and 5′ regulatory region of the *var2csa* gene was PCR-amplified and cloned upstream of a firefly *luciferase* reporter gene (pV2LH). A similar construct (pV2LHm) was made in which site directed mutagenesis was used to introduce a single base pair (bp) mutation that changed the start codon of the uORF from ATG to ACG, thus abolishing it as a start site for translation (Figure 1A). Transient transfection of these constructs into cultured parasites revealed that while pV2LHm showed reporter gene expression levels similar to those typically seen with other *var* promoters (pVLH), the intact uORF in pV2LH led to approximately 10-fold lower luciferase expression compared to pV2LHm (Figure 1B), confirming that this element strongly downregulates gene expression, potentially by repressing translation of the second ORF. Deletion of most of the 5′ regulatory region, leaving only 583 bp upstream of the *luciferase* start codon (pV2ΔLH), resulted in a construct that failed to produce any detectable levels of luciferase in our transient assays. This construct was used as the zero value in subsequent experiments, and indicated that despite the strong repression observed due to the presence of the intact uORF in pV2LH, complete silencing was not obtained and low-level luciferase expression remained detectable.

### uORF length correlates with extent of reporter gene repression

In most examples of uORF-regulated gene expression, repression of the downstream ORF occurs at the level of translation. Specifically, a scanning ribosome recognizes and initiates translation at the uORF, then terminates and dissociates from the mRNA either before or upon reaching the stop codon of the uORF. Thus the scanning ribosome is prevented from reaching the second ORF and expression of the encoded protein is prevented [29]. In some instances, expression of the second ORF can occur through a mechanism in which the ribosome continues scanning within the region separating the two ORFs and then re-initiates at the downstream ATG [34]. The efficiency of ribosomal re-initiation is influenced by both the length of the uORF and by the length of the intercistronic region (ICR), since a longer ICR allows the ribosome that has continued scanning an extended period to re-charge with initiation factors [35]. These properties are hallmarks of gene repression at the level of mRNA translation.

If the repression of reporter gene expression we observed in the pV2LH construct is due to a similar mechanism of translational repression, infrequent re-initiation might also be expected. To determine if the low-level luciferase expression observed from pV2LH is likely the result of re-initiation, the size of the uORF was manipulated without changing the length of the transcript leader. The uORF was shortened from 360 bp to 48 bp by introducing a stop codon (SURF) (Figure 1A). The effect was a 4-fold increase in luciferase expression compared to pV2LH containing an intact full-length uORF (Figure 1B). Conversely, the uORF was lengthened by deleting its stop codon, which effectively extended it to the next naturally occurring stop, making the uORF 450 bp long (uORFL). This further reduced luciferase expression. Thus, the extent of luciferase repression was strongly influenced by the length of the uORF, consistent with repression at the level of translation. However, increasing the length of the ICR by inserting an additional identical 260 bp sequence (ICL) had no apparent effect on reporter gene expression.

### The peptide encoded by the uORF is not required for repression

The coding region of the uORF is conserved in all *P. falciparum* isolates examined, as well as in *var2csa* of *P. reichenowi*, suggesting the possibility that the protein encoded by this ORF could play a role in repression of VAR2CSA expression. To test this possibility, we replaced this element with the similarly-sized *blasticidin-S-deaminase* (*bsd*) gene (pV2BLH) as well as with the longer *Renilla luciferase* gene (pV2RLH). Luciferase was strongly repressed in parasites transfected with both of these constructs (Figure 1B), indicating that the peptide encoded by the uORF is not required for repression. Parasites transfected with V2RLH showed strong expression of *Renilla luciferase*, demonstrating that the uORF itself is indeed translated (Figure 1C). The finding that the uORF supports efficient initiation of translation is consistent with uORF-mediated translational repression and with the presence of the Kozak consensus sequence, a motif important for translational initiation [36]. We took advantage of this fact to obtain a stably transfected line of pV2BLH, using *bsd* as a drug selectable marker.

### Repression via the uORF acts post-transcriptionally

**Figure 2:**
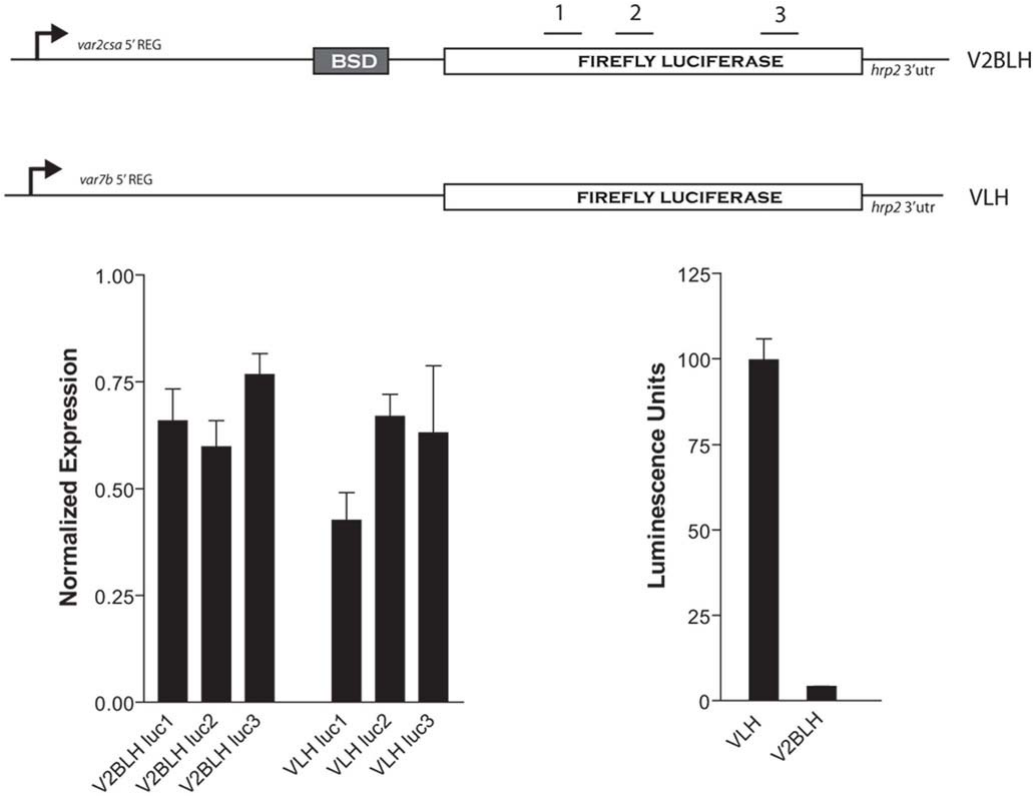
Translational repression in stably transformed parasites. Parasites were stably transfected with either pVLH or pV2BLH (A). V2BLH contains the *bsd* selectable marker and VLH contains *hdhfr* for maintaining the episomes in transfected parasites (not shown). Integration into the genome occurred spontaneously and was selected for by alternate growth with or without drug. *luciferase* mRNA levels were determined using Q-RT-PCR (B) with three different primer pairs (1–3), whose corresponding location on the *luciferase* ORF is shown in (A). Levels of protein expression were assayed by measuring luciferase activity (C).

The strong repression of reporter gene expression in constructs in which the *var2csa* promoter contained an intact uORF displayed many properties indicating that repression was acting at the level of protein translation. However, the possibility remained that an intact uORF in the *var2csa* upstream region instead simply reduced the rate of transcription from this promoter or produced an unstable mRNA. The pV2BLH construct allowed us to carry out a more detailed analysis of the effect of a uORF in a population of stably transformed parasites, and to specifically assess whether the repression was due to an effect on mRNA translation, the rate of transcription or mRNA stability. Parasites transfected with pV2BLH display comparable transcript abundance to those transfected with a similar construct in which the *luciferase* gene is driven by the heterologous *var*7b promoter as detected by real-time reverse transcriptase (RT) PCR (Figure 2B). However, pV2BLH showed 100 fold less luciferase expression (Figure 2C), demonstrating that in constructs containing an intact uORF, the RNA is actively transcribed and remains stable, however the second ORF, in this case encoding *firefly luciferase*, is not efficiently translated.

### Translational repression of *var2csa* in cultured parasites

**Figure 3:**
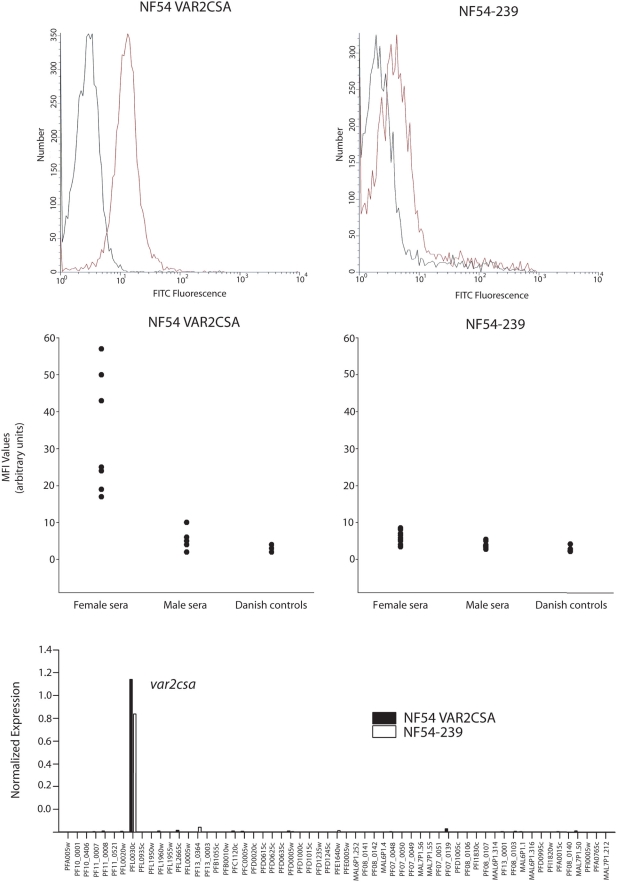
Evidence for translational regulation of *var2csa* in cultured, wildtype parasites. A. The parasite population NF54 VAR2CSA was selected using anti-VAR2CSA rabbit antibodies. Flow analysis indicates that a majority of the population displays VAR2CSA on the cell surface (red) with a MFI value of 14.2 compared to the background (black) with a MFI value of 5.2 (arbitrary units). Flow analysis of NF54-239 indicates that the majority of the population displays low levels of VAR2CSA on the cell surface with a MFI value of 5.0 compared to the background value of 3.6 (arbitrary units). B. Selection for VAR2CSA expression results in recognition by antibodies predominantly from female sera whereas the NF54-239 line is poorly recognized. C. *var* transcript analysis shows that *var2csa* is the dominant transcript in both the NF54 VAR2CSA (Black) and Nf54-239 (white) parasite lines.

All of the constructs that utilized the *var2csa* promoter and that included an intact uORF displayed substantial levels of repression of reporter gene expression, and this repression appeared to occur at the level of translation. However, as part of the functional design of these experiments, the coding regions of one or both ORFs had been replaced, raising the possibility that the observed repression could simply be an artifact of the design of the constructs. Therefore we attempted to identify similar translational repression in parasites in which the endogenous *var2csa* gene was actively transcribed. We had previously observed high levels of transcription of *var2csa* in cryo-preserved batches of NF54, which had not been selected (unpublished data). Further investigation of one of these parasite lines (NF54-239), using a real-time PCR primer set that examines transcription of the entire *var* repertoire of 3D7 [11], showed that *var2csa* was the overall dominant *var* transcript (Figure 3C). However, analysis of surface protein expression in these parasites using VAR2CSA-specific antibodies showed a very low but detectable uniform staining of the entire IE population (Figure 3A). The reactivity against the NF54-239 line using human serum was likewise low (Figure 3B). These data indicate that the majority of the parasites actively transcribe *var2csa*, but only express the protein at very low levels on the IE surface, suggesting that post-transcriptional repression similar to that observed in our reporter constructs may also regulate expression of the endogenous gene.

To determine if these parasites are capable of expressing VAR2CSA on the surface if placed under selective pressure, they were selected with VAR2CSA specific antibodies, and thus for active transcription and translation of VAR2CSA. As expected, the selected parasites (named NF54-VAR2CSA) were found to transcribe exclusively *var2csa* at high levels similar to the NF54-239 line (Figure 3C). However, unlike the unselected parasite population, flow cytometry using VAR2CSA-specific antibodies found uniform, intense staining of the entire IE population (Figure 3A), indicating that they were efficiently expressing VAR2CSA and trafficking it to the infected cell surface. The selected parasites also displayed predominant recognition by female-specific antibodies as well as binding to CSA (Figure 3B and data not shown).

PfEMP-1 expression at the RBC membrane can be reduced due to defects in protein trafficking or membrane presentation. The selection of a VAR2CSA expressing parasite population could thus be the result of selection of a subpopulation of NF54-239 parasites not deficient in PfEMP1 presentation. We used real time RT-PCR to examine expression of two genes known to play a role in these processes: *knob-associated histidine-rich protein* (Pf*kahrp*) and *skeleton-binding protein* (Pf*sbp*) [37],[38]. Both genes were present in the genomes of NF54-239 and NF54-VAR2CSA at equal levels compared to single-copy housekeeping genes. Both genes were also found to be highly transcribed in the non-VAR2CSA expressing NF54-239 line (data not shown), suggesting that lack of surface protein was not due to deficiency in either of these gene products.

### Expression of the downstream ORF

In other organisms that employ uORFs to regulate protein expression, the repressive effect of the uORF on translation of the downstream ORF can be overcome in response to some environmental cue. Since the *var2csa* uORF is present in transcripts isolated from parasites selected to actively translate the protein, indicating that it is not spliced from the message or otherwise altered, *P. falciparum* must also possess a mechanism for overcoming this type of translational repression. To determine if parasites might be responding to an environmental cue to overcome the translational repression of the uORF in the *var2csa* upstream region, parasites transfected with pV2BLH were incubated in a variety of chemicals and nutrients thought to be found in high concentrations in syncytiotrophoblasts. However, the addition of soluble CSA, hyaluronic acid, progesterone and cortisol, spermine and spermidine, amino acids and glucose all failed to induce increased expression of *luciferase* (data not shown).

**Figure 4:**
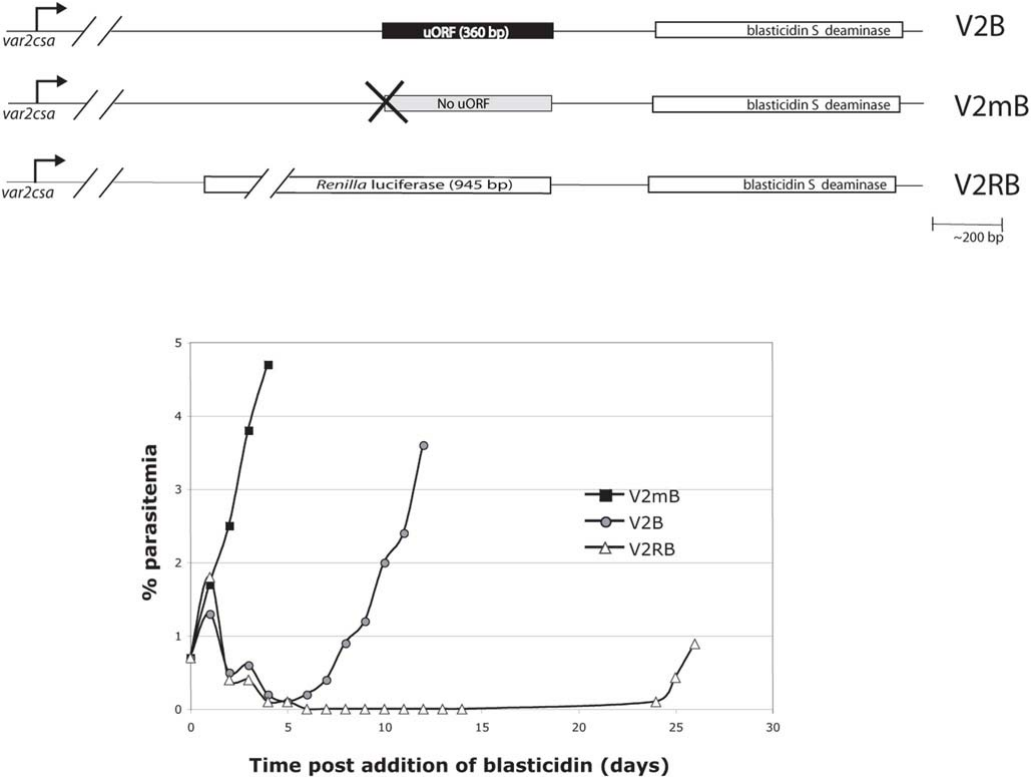
Selection for reversal of translational repression by the uORF of *var2csa*. A. Constructs used for selection of parasites that translate the second open reading frame of the *var2csa* gene. The drug selectable marker *blasticidin-S-deaminase* (BSD) was used to select parasites translating the downstream cistron (equivalent to exon I of *var2csa*). All three constructs also contain the *hdhfr* selectable marker for maintaining the episomes in transfected parasites prior to blasticidin selection (not shown). B. A drug resistant population appeared after six days suggesting that a subset of parasites is capable of translating the second ORF. Replacing the uORF with *Renilla luciferase* (V2RB) led to severely retarded growth in presence of blasticidin. Analysis of episomes recovered from these parasites indicated that they had undergone recombination (not shown).

**Figure 5:**
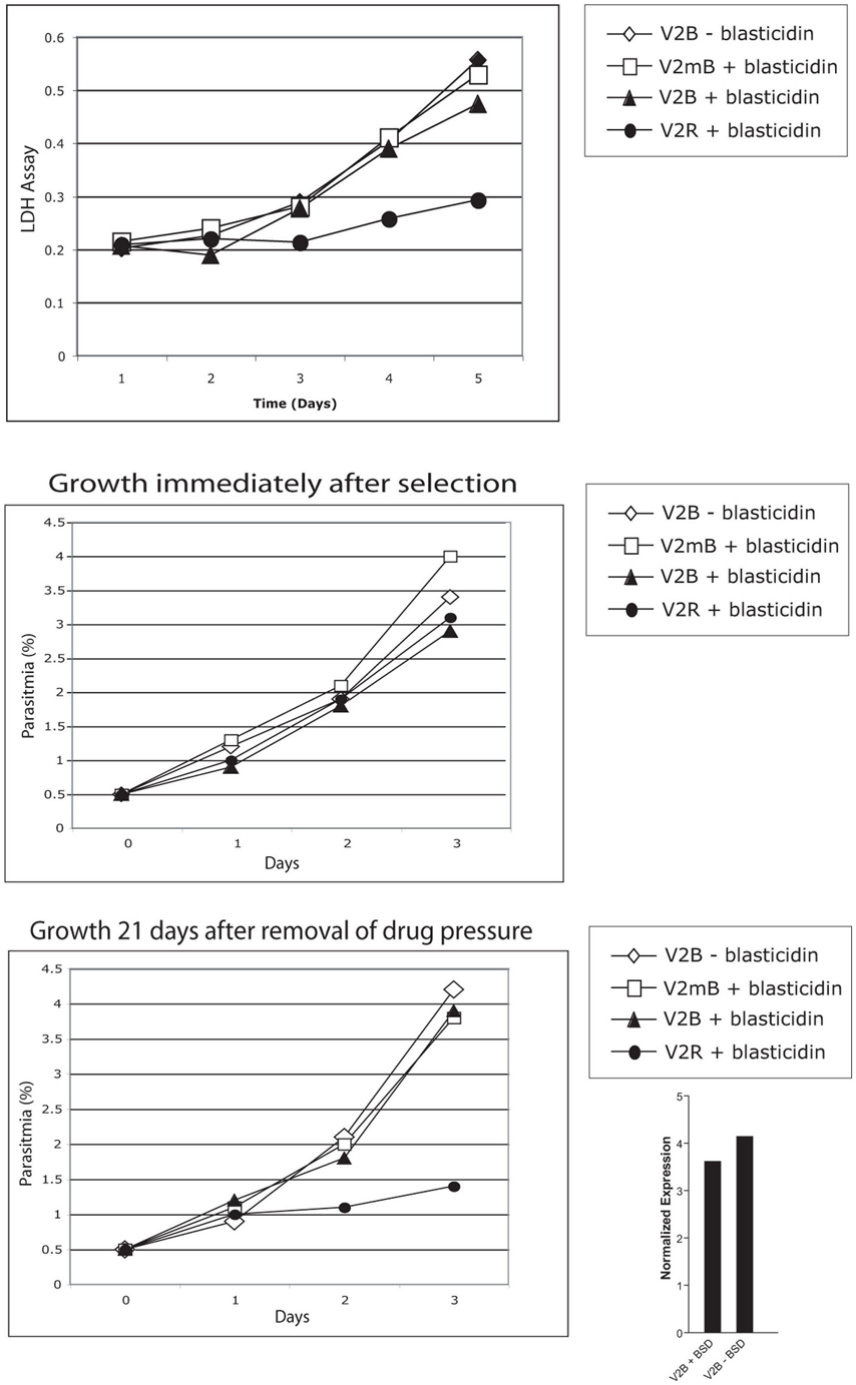
Growth rates of parasites translating different ORFs. Constructs are shown in Figure 4. A. Parasites that have been selected for translation of the second open reading frame encoding *blasticidin-S-deaminase* (V2B+blasticidin) grow at the same rate as parasites not under blasticidin selection (V2B−blasticidin) or those in which translation of the uORF has been disrupted (V2mB+blasticidin). Parasites in which the uORF has been replaced with the coding region of *Renilla luciferase* (V2RB+blasticidin) grow at a slower rate. B. Parasites actively translating the second ORF encoding *blasticidin-S-deaminase* continue to display resistance to the drug if grown without selection pressure very briefly. C. When parasites are grown in the absence of blasticidin in the media for 21 days, they rapidly begin to repress translation of the second ORF and revert to the drug sensitive phenotype as demonstrated by slower growth when placed back under drug pressure (V2B+blasticidin). The histogram at the right shows that reversion to drug sensitivity is not due to a switch in promoter activity. Parasites grown without blasticidin pressure (V2B−BSD) for three weeks continue to express *bsd* mRNA at levels equal to or greater than those grown continuously under blasticidin pressure (V2B+BSD). *bsd* mRNA levels are shown as copy number normalized to *seryl-tRNA synthetase*.

Instead of responding to an environmental cue, it is also possible the parasites stochastically commence translating the second ORF, and that selection for VAR2CSA surface expression, for instance by “panning” cultured parasites, simply enhances this population. The fact that cultured parasites can be selected to actively express VAR2CSA without any changes to the culture media is consistent with the idea that an environmental cue is not required to induce expression of the second ORF. To test this model, we utilized a different strategy in which we placed a drug-selectable marker in the place of the second ORF (corresponding to the VAR2CSA coding region) to select a population of cells that actively translate the downstream ORF. We constructed three different plasmids: pV2B contained a *var2csa* 5′ UTR with an intact uORF; pV2mB contained a single base-pair mutation that abolished the uORF and pV2RB had *Renilla* luciferase in place of the uORF (Figure 4A). These constructs were stably transfected into cultured parasites and maintained as episomes. When the parasites were challenged with blasticidin, we observed the following: parasites transfected with pV2mB, in which the uORF was mutated, continued growing at a normal rate, indicating they were resistant to blasticidin and thus that the drug resistance gene was actively expressed (Figure 4B). Parasites transfected with pV2B failed to show any growth for the initial 3–5 generations after the addition of blasticidin, however a population of parasites subsequently arose that grew at a normal rate. Recovery of episomes from these parasites indicated that that uORF remained intact in the constructs (data not shown). We interpret this as selection of a sub-population of parasites that have begun to efficiently translate the downstream ORF. pV2mB and pV2B grew at similar rates (Figure 5A), a finding that rules out the possibility that the uORF simply diminishes the rate of translation of the drug resistance gene as this would be reflected in a reduced growth rate. Rather, a true switch in translational efficiency seemed to be operating. Thus parasites demonstrate two distinct states: a translation-repressed population and a translation-competent one.

For pV2RB transfected parasites, growth was undetectable for several weeks but parasites did eventually re-appear in the culture (Figure 4B), however they grew at much slower rates (Figure 5A). Plasmid rescue revealed that pV2B and pV2mB episomes were intact without any deletions or rearrangements, however episomes recovered from pV2RB were extensively rearranged (data not shown), suggesting that parasites might have deleted or otherwise inactivated the *Renilla luciferase* ORF. This sequence is much larger than the uORF and this is potentially one reason why we were unable to select for expression of BSD. Alternatively, the endogenous uORF may be the only sequence that permits translation of the downstream cistron. This is further supported by data obtained in our original transient transfections, where pV2LH (with an intact uORF) always demonstrated a low level of luciferase activity, whereas pV2BLH (*bsd* instead of uORF) gave values closer to zero.

### The ability to translate the downstream ORF is transient

In order to test if the switch to translation of the downstream ORF is a stably inherited event, V2B parasites selected for growth in blasticidin were taken off drug pressure for a period of three weeks. Upon subsequent re-challenge with blasticidin, V2B parasites again demonstrated a delayed growth phenotype (Figure 5C) indicating they had stopped translating the second ORF in the absence of drug pressure and switched back to the translationally repressed state. Growth of these parasites without blasticidin pressure for greater than one month did not affect *bsd* transcript levels, implicating repression of translation in the loss of blasticidin resistance. Continuous selection therefore appears to be necessary to ensure expression of the major ORF of the *var2csa* transcript.

## Discussion

Translational repression has been identified as an important regulatory mechanism in the sexual development of Plasmodium. In female gametocytes, several transcripts are stored in translationally-repressed cytoplasmic bodies. These transcripts contain specific cis-acting sequences [39] and are maintained at steady-state levels until blood-stage gamete precursors are ingested by a mosquito, at which point they can be translated [40]. Other instances of translational regulation in Plasmodium have not yet been described, but are likely to exist considering the relative scarcity of specific transcription factors in this organism [41]. The use of uORFs, like that described here for the *var2csa* transcript, is one common way of regulating translation. Functional uORFs have been associated with genes that control cellular growth in various organisms [42], including several human oncogenes [43],[44]. Some studies predict the occurrence of functional uORFs to be as high as 25% in mammalian genes [45] and they have been identified in several hundred fungal genes [46],[47]. Bioinformatic studies in different species are faced with the difficulty of distinguishing between functional uORFs and spurious ones that appear by chance alone. Without direct mutational analysis of individual uORFs, studies must rely on conservation as a predictor of functionality. The *var2csa* uORF in *P. falciparum*, having been demonstrated to be a functional translational repressor, is 94% identical at the sequence level to the uORF in the *P. reichenowi* ortholog, but this is approximately the same level of conservation observed for the entire 5′ UTR, making it difficult to determine if the amino acid coding potential is under selection. uORFs in Drosophila are predicted to have a mean length of 70 amino acids [48] while in yeast they are thought to be only 4–6 codons in length [47]. This makes the 120 amino acid *var2csa* uORF unusually large. Other uORFs in *Plasmodium* genomes have not been described and their frequency has not been analyzed.

uORFs generally act as translational repressors. Several mechanisms have been described for how they inhibit translation of the downstream ORF. For example, in some instances ribosomes stall on the uORF, and this is typically a sequence-dependent interaction with the nascent peptide [49],[50]. This seems to be an unlikely mechanism of repression in the case of *var2csa* since the amino acid sequence of the uORF does not appear to be required for repression. Other uORFs initiate nonsense mediated decay (NMD) by mimicking pre-termination codons [33], however this also seems to be unlikely for *var2csa* since steady state mRNA levels transcribed from our reporter constructs appear to be stable. In addition, cultured parasites that transcribe the endogenous *var2csa* gene but do not translate the encoded PfEMP1 also display stable mRNA levels. In a third mechanism of repression, scanning ribosomes translate the uORF and then fail to re-initiate translation at the downstream ORF. This seems to be the most likely scenario for the repressive effect of the uORF in *var2csa*.

Correspondingly, different mechanisms exist for overcoming the repressive effect of uORFs and activation of translation of downstream ORFs. In the case of Neurospora *arg-2*, reduced arginine levels lead to bypassing of the uORF by leaky scanning [50], thus translation preferentially initiates at the start codon of the second ORF. In other examples, translation of the second ORF depends on the ribosome reinitiating a second round of translation upon reaching its start codon. When re-initiation is the mechanism by which the downstream cistron is translated, the sequence of the uORF stop codon, the intercistronic region (ICR) and the phosphorylation status of initiation factors in the cell are thought to be important for re-initiation efficiency [29]. This type of mechanism can be identified by manipulation of the size of the uORF and the ICR. Artificially lengthening the ICR will increase the levels of re-initiation because the ribosome will have more time to re-charge as it continues scanning towards the downstream initiation codon [35]. Finally, internal ribosome entry sites (IRES) [51] or ribosome shunts [52] can be used to avoid the repression caused by the uORF.

Due to the unusual length of the *var2csa* uORF, it is uncertain whether a re-initiation mechanism could lead to translation of the VAR2CSA-coding ORF since re-initiation efficiency is known to diminish with increased size of uORFs. In fact, in yeast, increasing the size beyond 35 codons effectively drove re-initiation efficiency to zero [53]. Transient transfections indicate that changing the length of the ICR has no effect on expression of the downstream reporter (Figure 1), which also argues against a re-initiation mechanism. However, increasing the length of the uORF reduced luciferase expression while shortening it led to increased activity, a finding that is reminiscent of re-initiation mechanisms. Direct examination of ribosome behavior on the transcript leader will be necessary in order to determine if re-initiation is occurring. Similarly, the presence of an IRES element has not been tested in this study.

The uORF-mediated repression of translation described here provides a mechanistic explanation for the repression of *var2csa* described by Mok et al. [27]. In both studies, parasite lines were identified in which *var2csa* was shown to be sparsely translated despite abundant mRNA production. Furthermore, in both studies the infected cells were depleted of PfEMP1 because translational repression of VAR2CSA was not accompanied by activation of expression of another PfEMP-1 molecule, as evidence by lack of recognition by immune sera. Mok et al. described frequent switching to transcription of *var2csa* by unselected parasites and hypothesized that this represents a default state of *var* gene expression. While in the current study we did not examine switching within the *var* repertoire, previous work in our laboratory failed to detect any significant switching to *var2csa* within clonal populations [54]. However we have frequently detected *var2csa* transcription in the uncloned NF-54 line, which may be related to the “off-switching” described by Mok et al.

Despite the presence of the repressive element, *var2csa* mRNA does in fact get translated, most notably during infection of pregnant women, but also in culture after selection for binding of cells to CSA. The uORF could function to ensure that VAR2CSA protein is only rarely expressed by parasites during an infection. Thus, in addition to low-frequency transcriptional activation (switching), only a subset of parasites actively transcribing *var2csa* would be synthesizing VAR2CSA protein. This subpopulation would then expand by selection when the cognate receptor is present to allow binding and sequestration of the infected cells. This type of mechanism implies stochastic activation of translation, and VAR2CSA would be much less frequently expressed than PfEMP1s encoded by other members of the *var* gene family. A potential advantage for the parasite of repressing VAR2CSA translation in the absence of its binding niche is the avoidance of immune memory which would compromise the utility of this receptor. Indeed, most studies have shown that antibodies against placental-binding IEs are undetectable in individuals who were never pregnant [16]–[19], contrary to what one might expect in individuals raised in endemic areas where they are continuously exposed to parasites with random *var* expression patterns. Fried and colleagues did manage to detect peptides corresponding to VAR2CSA in infected erythrocytes from children [25], however this might represent the product of low level translation, similar to that observed in our translationally-repressed parasites. Further studies into the mechanisms used by malaria parasites to regulate VAR2CSA expression, as well as protein expression in general, will provide greater insights into the molecular basis of the host/parasite interactions that underlie the pathogenesis of the disease.

## Materials and Methods

### Plasmid Constructs

pV2LH was derived from the pHLH (previously described) [55]. The *var7b* promoter was replaced with the *var2csa* promoter that was amplified from NF54 parasites using the following primers: 5′-GGTACCTGAACGCTTAAAGAAACAAGG-3′ and 5′-CTGCAGCATTTTGTCCAACCATTTACA-3′ . V2LHm was obtained by performing site-directed mutagenesis on V2LH using Stratagene's Quickchange kit and the primer 5′-GACATATAACCACGGAATCAGAGTATCAAAAACAAACATCTATAGG-3′. V2BLH was obtained by replacing the *var2csa* uORF with the *blasticidin-S-deaminase* gene obtained from VBbIDH [56]. V2ΔLH was obtained by digesting V2LH with Kpn I and self-ligating the backbone. The V2BbIDH series was obtained by modification of VBbIDH [56].

### Parasite Culture and Transfection

NF54-239 was received from the Division of Medical Parasitology and Centre for Clinical Malaria Studies, Radboud University Nijmegen, The Netherlands. The NF54-VAR2CSA line was selected for VAR2CSA expression as described [12]. The parental line of both these lines was first described by Ponnudurai et al. [57]. *P. falciparum* parasites were cultivated at 5% hematocrit in RPMI 1640 medium, 0.5% albumax II (Invitrogen), 0.25% sodium bicarbonate, and 0.1 mg/ml gentamicin. Cultures were maintained at 37°C in an atmosphere of 5% oxygen, 5% carbon dioxide, and 90% nitrogen. Reporter constructs were transfected into the NF54 parasite line by using the “DNA-loaded” red blood cell method as described previously [58]. Briefly, 0.2-cm electroporation cuvettes were loaded with 0.175 ml of erythrocytes and 50 µg of plasmid DNA in incomplete cytomix solution. For stable transfection, NF54 parasites were cultured in media containing 40 ng/ml pyrimethamine or 2 µg/ml Blasticidin S HCl (Invitrogen). Plasmid rescue experiments were performed by transforming *Escherichia coli*-competent cells with 500 ng of purified *P. falciparum* genomic DNA.

### RNA Extraction and Realtime RT-PCR analysis of gene expression

RNA was extracted from synchronized ring stage parasites 16–18 h post-invasion. RNA extraction was performed with the TRIZOL LS Reagent (Invitrogen) as previously described [59]. RNA was purified using RNeasy MiniElute columns (Qiagen) according to manufacturer's protocol. Isolated RNA was then treated with Deoxyribonuclease I (Invitrogen) to degrade contaminating gDNA. cDNA synthesis was performed with Superscript II Rnase H reverse transcriptase (Invitrogen) with random primers (Invitrogen) as described by the manufacturer. 800 ng of total RNA was used for each cDNA synthesis reaction. A control reaction without reverse transcriptase was performed with identical amounts of template. To quantify *luciferase* transcription levels we used three primer pairs that hybridized to different regions of the cDNA molecule: 5′ GCTGGGCGTTAATCAGAGAG 3′ and 5′ ACTGGGACGAAGACGAACAC 3′, 5′ CGGATTACCAGGGATTTCAG 3′ and 5′ CAGGCAGTTCTATGCGGAAG 3′, 5′ TGTTGTTCCATTCCATCACG 3′ and 5′ CAGAGTGCTTTTGGCGAAG 3′. To quantify *blasticidin-s-deaminase* we used the primers: 5′-TTGTCTCAAGAAGAATCCAC-3′ and 5′-TCCCCCAGTAAAATGATATAC-3′. To quantify *kahrp* and *sbp1* transcription levels we used the following primers pairs: *kahrp*: 5′ TCACCAACAAGTACATGGTCAA 3′ and 5′ ATATGCTTTGAAACCTCCACCT 3′ , *sbp1*: 5′ GCAACGTAGAATGGCTCAAG 3′ and 5′ ACATGTACGGCTTGTTTTGC 3′.

All runs were done in triplicate and copy number was determined using absolute quantization by interpolation from standard curves obtained from 10-fold serial dilutions of either genomic DNA (for *luciferase* and *seryl tRNA synthetase*) or linearized plasmid (for *blasticidin-s-deaminase*) (User bulletin 2, Applied Biosystems, http://www.appliedbiosystems.com). The primer amplification efficiency was calculated on the basis of real-time measurements of 10-fold dilutions of DNA. We used (e) = 10∧(−1/slope)−1 to verify the efficiency of the primers. Expression was normalized to amount of control gene *seryl tRNA synthetase*, in order to ensure comparison of equal amounts of cDNA.

Reactions were performed at a final primer concentration of 0.5 µM using Biorad ITAQ SYBR green Supermix in 20-µl reactions on an ABI Prism 7900HT. To quantify *var* gene expression levels in NF54-VAR2CSA and NF54-239 we employed the methods and primers described in Dahlback et al [60]. Absolute quantification of the copy number of each individual gene was based on standard curves derived from serial dilutions of gDNA.

### Flow Cytometry

Parasite cultures were enriched to contain >75% erythrocytes infected by late trophozoite and schizont stage parasites by exposure to a strong magnetic field [61]. Aliquots (2×10^5^ IE) were labeled by ethidium bromide (2 µg/ml) (to allow exclusion of remaining uninfected erythrocytes). Surface staining of IE was done with DBL5-VAR2CSA specific rabbit serum and mock immunized rabbit serum as control [12]. Samples were sequentially exposed to; 20 µl rabbit serum pre-absorbed against uninfected erythrocytes; 1 µl biotinylated sheep-anti-rabbit IgG (The Binding Site) and 0.5 µl streptavidin-FITC (BD Pharmingen). For IE surface staining with human IgG, samples were sequentially exposed to 5 µl plasma, and to 1 µl goat–anti-human IgG (Vector, Burlingame, US) in a total volume of 100 µl. A panel of plasma samples from *P. falciparum* exposed adult males and females, and non-exposed Danes were used to test the parasites for gender specific recognition. All incubations were performed in a total volume of 100 µl PBS with 2% FCS for 30 min. Samples were washed three times with 200 µl PBS with 2% FCS between each incubation. Data from a minimum of 5000 IEs were acquired using a FC500 flowcytometer (Beckman Coulter). Flow cytometry analysis was repeated two times on different parasite preparations with similar results.

### Lactate Dehydrogenase Assay

*In vitro* parasite growth was measured by an adaptation of a method developed by Goodyer et al. [62]. 30 µl of culture was centrifuged, lysed by freeze-thawing and resuspended in 15 µl of PBS. 10 µl of this solution was placed into a well of a 96-well plate. Each well then received 100 µl of a solution consisting of 0.1% Triton X-100, 10 mg/mL L-lactic acid, 3.4 mg/mL Tris-HCl and 0.34 mg/mL 3-acetylpyridine adenine dinucleotide at pH 9, as well as 20 µl of a mixture of 1 mg/mL Nitro Blue Tetrazolium and 0.5 mg/ml Diaphorase. The plate was covered with aluminum foil and shaken for 15–25 minutes after which the reaction was stopped with 75 µl of acetic acid. Colorimetric measurements were made at 650 nm.
